# Recent Advances in In Vitro and In Vivo Studies of Antioxidant, ACE-Inhibitory and Anti-Inflammatory Peptides from Legume Protein Hydrolysates

**DOI:** 10.3390/molecules28062423

**Published:** 2023-03-07

**Authors:** Deia Tawalbeh, Muhammad H. Al-U’datt, Wan Amir Nizam Wan Ahmad, Fisal Ahmad, Norizah Mhd Sarbon

**Affiliations:** 1 Faculty of Fisheries and Food Science, Universiti Malaysia Terengganu, Kuala Nerus 21030, Terengganu, Malaysia; 2 Department of Nutrition and Food Technology, Faculty of Agriculture, Jordan University of Science and Technology, P.O. Box 3030, Irbid 22110, Jordan; 3 School of Health Sciences, Universiti Sains Malaysia, Kubang Kerian 16150, Kelantan, Malaysia

**Keywords:** legumes, isolate, hydrolysate, bioactive peptides, in vitro, in vivo studies

## Abstract

Consumption of legumes has been shown to enhance health and lower the risk of cardiovascular disease and specific types of cancer. ACE inhibitors, antioxidants, and synthetic anti-inflammatories are widely used today; however, they have several undesirable side effects. Thus, researchers have focused on finding ACE inhibitors, antioxidant, and anti-inflammatory peptides from natural sources, such as legumes. Recently, in vitro and in vivo research has shown the bioactive peptides generated from legume protein hydrolysates, such as antioxidant, anti-hypertensive, anticancer, anti-proliferative, anti-inflammatory, etc., in the context of different disease mitigation. Therefore, this review aims to describe the recent advances in in vitro and in vivo studies of antioxidant, anti-hypertensive and anti-inflammatory peptides isolated from legume-derived protein hydrolysates. The results indicated that antioxidant legumes peptides are characterized by short-chain sequence amino acids and possess anti-hypertensive properties by reducing systolic blood pressure (SBP) in spontaneously hypertensive rats (SHR).

## 1. Introduction

Legumes are edible seeds of plants belonging to the Leguminosae family and are considered the third-biggest flowering plant family, with 946 genera and 24,505 species [1]. Legumes, such as soybeans, peas, chickpeas, lentils, beans, and peanuts, are considered a rich source of protein (30–35%), and are an inexpensive and popular food for many people [2]. Legumes are widely grown and consumed worldwide because they play an essential role in the human diet. Their rich sources of protein, minerals, carbohydrates, and vitamins are related to the prevention of chronic diseases due to their contents of bioactive peptides [3]. Therefore, legume protein hydrolysates have been widely studied to produce peptides with biological properties such as antihypertensive, antioxidant, antiproliferative, anticoagulant, anti-inflammatory, anticancer, hypoglycemic, calcium-binding, immunomodulatory, and anti-obesity [4,5,6].

The production and quality of protein from legumes are impacted by extraction techniques [7]. One of the common processes, referred to as isoelectric precipitation, that is used to extract isolated protein from legumes depends on the solubility. The pH ranges of alkaline and acid lead to high solubility, while the isoelectric point (around pH 4–5) exhibits low solubility [8]. Because alkaline solutions can accelerate the dissolution of protein from legumes, several legumes are employed to extract protein using alkaline solutions (NaOH) [7], such as lupine [9], chickpea [10], black bean [11], soy [12], and pigeon pea [13]. In addition, Langton et al. [14] mentioned that the extraction conditions such as pH, choice of solvent, protein fractions and other compounds in protein isolate, and temperature can affect the characteristics of the protein extracted from legumes. In contrast, enzymatic hydrolysis increases the rate at which protein is extracted by hydrolyzing the protein, enhancing protein solubility, and rupturing the legume cell wall, which encourages the solvent to penetrate the tissue of the legumes, thereby increasing the protein dissolution [7]. Besides, enzymatic hydrolysis is the most popular method for generating hydrolysate and bioactive peptides from legumes [15,16]. Recently, enzymatic hydrolysis of the legume proteins has been reported as an effective, cheaper, and safe method to release bioactive peptides with specific health benefits, such as antioxidant properties and antihypertensive (inhibitors for the angiotensin-I converting enzyme (ACE) [17].

Bioactive peptides may be considered edible nutraceutical agents with health benefits related to disease treatment or prevention [18]. Throughout the past few decades, many studies have focused on the isolating and purifying of antioxidant and ACE-inhibiting peptides of legume protein hydrolysates such as lentils [19], black bean [20], mung bean [21], chickpea [22], soybean [23], and pigeon pea [24]. Synthetic ACE inhibitors have various adverse side effects, while synthetic antioxidants are banned due to health risks [25]. On the other hand, antihypertensive and antioxidant peptides derived from natural plant and animal sources such as tuna muscle, wheat, casein, soy, eggs, milk, azufrado beans, cheese, fish, meat, corn gluten, chickpea, peanut soybean, lupin, hemp, pea, lentil, Bambara, and common bean have potentially beneficial effects due to their high activity, low cost, easy absorption in the human body, and low molecular weight. In addition, they have little or no adverse side effects and strong antioxidant activities [26]. Wang et al. [27] stated that antioxidant peptides are shown to have ACE-inhibitory activity concurrently. The ACE-inhibitory and antioxidant peptides have been deeply related to their structure, sequencing, chain length, and amino acid composition [28]. In addition, Wang et al. [29] mentioned that the presence of hydrophobic amino acids (Cys, Pro, Leu, Met), aromatic amino acids (Trp, Phe, and Tyr), positively charged amino acids (Arg and Lys), and branched-chain aliphatic amino acids (Val, and Ile) is a typical characteristic of peptides with high ACE-inhibitory activities.

Studies of the ACE-inhibitory, antioxidant, and anti-inflammatory bioactive peptides in legumes have been conducted in vitro, with only a few studies providing confirmatory results in vivo. In an in vitro study, Garcia-Mora et al. [30] found that pinto bean protein hydrolysate has potent ACE inhibitory (IC_50_: 0.22–0.26 mg/mL) and free radical scavenging (326–348 mmoLTrolox eq./g) with small peptides <3 kDa. These peptides are used as a valuable component in developing healthy foods. Furthermore, according to Márquez et al. [31], peptide fractions of chickpea protein hydrolysate have anti-inflammatory activity by reducing levels of nitric oxide inhibition (F5–10 kDa: 76%), interleukin-1 (F3–5 kDa: 90%), and tumor necrosis factor (F3–5 kDa: 93%). In contrast, some of these bioactivities have been proven in vivo, confirming their physiological importance [32]. According to a study by Valenzuela-García et al. [33], the IC_50_ of the ACE-inhibitory activity of Azufrado bean protein hydrolysate was 3.68 µg/mL, which showed an antihypertensive impact on spontaneously hypertensive rats (SHR) as short and long periods, respectively (−41 mmHg, −24 mmHg). Consequently, the results from in vitro and in vivo studies are helpful to food scientists and could substantially impact human health by preventing or controlling high blood pressure. Therefore, this paper reviews recent advances in in vitro and in vivo studies on antioxidant and ACE-inhibitory peptides isolated from legume protein hydrolysates.

## 2. Legume Protein Isolate and Hydrolysate

The extraction of protein isolates and other components from legume seeds is currently performed using advanced legume extraction and fractionation technologies. Protein isolation can be done in two ways: dry processing for legume seeds, which preserves protein functionality, and wet processing for flours, which yields better protein purity [34]. Previous studies have reported on legume protein isolation using the acid-base extraction method, such as chickpea [35], black gram [36], pea [37], pinto beans [38], string beans [39], and lupin [40]. The legume protein isolation begins with extracting the protein in an alkaline solution, followed by isoelectric precipitation (IP), and finally, drying to get the final isolated protein [41,42]. Los et al. [43] extracted carioca bean protein before hydrolysis with NaOH (pH 8.0) and then precipitated it with HCl (pH 4.5).

Legume protein hydrolysate can be produced using numerous methods, such as chemical hydrolysis, enzymatic hydrolysis, or microbial fermentation [38,44]. Enzymatic hydrolysis converts the protein molecules into peptides quickly for various sizes and free amino acids by breaking down certain peptide bonds of the parent protein using proteases with a positive impact on human health [45]. In this context, through the extraction of protein isolate, enzymatic hydrolysis, chemical and fermentation synthesis, separation, and purification (ultrafiltration membrane, gel filtration, reverse phase high performance liquid chromatography, and ion exchange chromatography), various legume sources can be converted into antioxidants, ACE-inhibitory, and anti-inflammatory peptides [2,23,31]. These peptides are regarded as a good source and an alternative food for use as antioxidants, antihypertensive, and anti-inflammatory peptides [46,47,48]. Moreover, antioxidant and ACE-inhibitory properties of legume protein hydrolysates are strongly influenced by hydrolysis, which changes the structural and composition characteristics such as molecular weight (MW) [11,49,50]. Putra et al. [13] mentioned that peptide fractions with MW below 1 kDa demonstrated the highest ACE-inhibitory activity among the other fractions derived from pigeon pea hydrolysates because of their capacity to bind Zn^2+^ and form Peptide- Zn^2+^ complex, which prevents ACE from using the Zn^2+^ ions that are already present as a cofactor. According to Chen et al. [51], antioxidant peptides were separated from the black bean soybean protein hydrolysate using ultrafiltration, gel filtration (GF), and reverse phase high performance liquid chromatography (RP-HPLC). The amino acid sequence of the sub-fraction (F2-c) peptide (455 Da) was identified as Leu-Val-Pro-Leu-Lys and Ile-Val-Pro-Leu-Lys and has the highest antioxidant of DPPH and HRSA (IC_50_: 0.12 µM and 0.037 µM).

## 3. In Vitro and In Vivo Studies on the Antioxidant, ACE-Inhibitory, and Anti-Inflammatory Peptides from Legume Protein Hydrolysate

### 3.1. In-Vitro Study of Antioxidant, ACE-Inhibitory, and Anti-Inflammatory Peptides

#### 3.1.1. Antioxidant Peptide

Reactive oxygen species “(ROS)” are highly m molecules produced by all aerobic cells that can be free radicals, such as hydroxyl radical(•OH), and superoxide radical(•O_2_) or non-radicals, such as singlet oxygen(^1^O_2_) and hydrogen peroxide(H_2_O_2_) [52]. Free radicals are unavoidable metabolic by-products that are the result of oxidative stress which can damage proteins, cell membranes, phospholipids, and DNA, resulting in severe human diseases such as diabetes, coronary heart disease, hypertension, stroke, cancer, arteriosclerosis, and Alzheimer’s [53,54]. Therefore, antioxidants play a crucial role in preventing or delaying the autoxidation of food components and the human body by inhibiting oxidation reactions and producing free radicals [55]. Antioxidant peptides released during enzymatic hydrolysis act as free radical scavengers, metal inactivators, oxygen inhibitors, or peroxide to protect the body and food system from reactive oxygen species [56]. Antioxidants are classified into synthetic and natural categories [57]. Although synthetic antioxidants such as “butylated hydroxytoluene (BHT), propyl gallate (PG), butylated hydroxyanisole (BHA), and tert-butyl hydroquinone (TBHQ)” are efficient and relatively cheap, they have displayed some toxic and hazardous properties [58]. According to Lourenço et al. [59], several studies have been published showing a correlation between the long-term consumption of synthetic antioxidants and specific health problems such as digestive system disorders, skin allergies, and occasionally leading to increased risk of cancer.

Additionally, in animal studies, BHT and BHA have already been shown to be responsible for adverse effects on carcinogenesis and the liver. Large dosages of synthetic antioxidants may cause premature senescence and damage the DNA. For this reason, studies have focused on searching for and developing antioxidant peptides from natural sources such as plants and animals [60]. These natural antioxidant peptides can be easily absorbed more readily and without adverse side effects compared to synthetic antioxidants. Furthermore, the primary mechanism of these antioxidant peptides is through the ability to inactivate intracellular reactive oxygen species (ROS), scavenge free radicals, reduce lipid peroxidation, and chelate transition metals, among other things, which have all been linked to their antioxidant activities [61].

Nevertheless, compared to synthetic antioxidants, some natural antioxidants have lower antioxidant activity, indicating that they need to be used in higher dosages and may result in unsafe dosages. Despite this, if they are taken within the limits of the law, natural antioxidants are a helpful alternative to synthetic ones [59]. Food-derived antioxidant peptides are considered natural antioxidant resources which possess acceptable nutritive value, minimal adverse side effects (safe), high efficiency, low molecular weight, high activity, low cost, and are easily absorbable to replace synthetic antioxidants for use in food [52,62].

**Figure 1:**
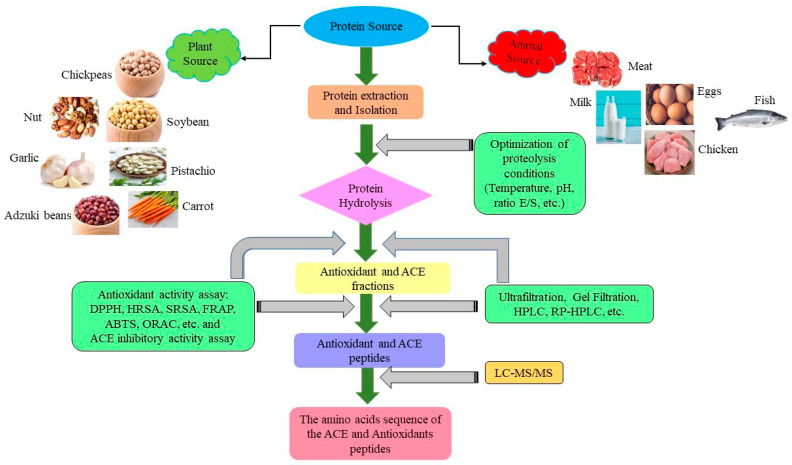
Schematic representation of the production of antioxidants and ACE peptide from natural protein sources.

Many studies have shown that legumes, such as chickpeas, pigeon peas, beans, soybeans, and lentils, can produce antioxidative peptides in vitro and positively impact human health when used as alternative antioxidants [3]. The antioxidant activity of peptides depends on molecular weight, amino acid composition and sequence, size, hydrophobicity, enzyme specificity, and degree of hydrolysis [20,46]. Figure 1 shows the main steps for preparing and identifying antioxidant peptides from natural protein sources. In this context, if an antioxidant compound prevents the generation of free alkyl radicals or disrupts the free radical chain’s propagation, the lipid oxidation’s chemical reaction rate is delayed or slowed. The use of singlet oxygen inhibitors, metal-chelating agents, and peroxide stabilizers caused this delay by donating hydrogen from antioxidants and metal-chelating agents [63]. Recent interest in antioxidant peptides derived from food proteins has grown owing to their prominent role in the prevention mechanisms of oxidative stresses linked to various diseases [64]. Consequently, different methods of in vitro antioxidant systems have been used to assess the antioxidant capacity of legume protein hydrolysates and peptides such as (DPPH) 2,2-diphenyl-1-picrylhydrazyl, (HRSA) hydroxyl radical scavenging activity, (ABTS) 2,2-azino-bis-3-ethylbenzothiazoline-6-sulfonic acid radical scavenging, and (ORAC) oxygen radical absorbance capacity, etc. [65,66]. Lentil protein hydrolysate with an amino acid sequence Ala-Leu-Gly-Pro-Val-Met (587.31 Da) exhibited the highest DPPH (63%) and β-carotene-linoleate model system (73%), respectively [65]. Wali et al. [67] also mentioned that the identified antioxidant peptide of chickpea protein hydrolysate (NF2-4-1): Leu-Thr-Glu-IIe-IIe-Pro (685.41 Da) showed the highest DPPH and OH scavenging activities, IC_50_: 0.24 mg/mL and 0.57 mg/mL, respectively. Peptides with low molecular weight have strong antioxidant activities than higher molecular weight peptides because they have a better chance to cross the intestinal barrier to exert antioxidant effects and favor increased interactions with the free radical [68]. Table 1 summarizes the in vitro antioxidant activities of legume protein hydrolysates and peptides.

The presence of hydrophobic (Pro, Val, Leu, Ala, Tyr, Trp, Met), basic (Arg, His), and aromatic (Trp, Phe, Tyr) amino acid residues in the peptide sequence can contribute to increasing antioxidant activities [42]. Phongthai and Rawdkuen [58] clarified that Val and Ile might be responsible for generating a suitable hydrophobic microenvironment for peptide molecules, while indole and pyrrolidine rings in Pro and Trp, respectively, could also act as hydrogen donors via their hydroxyl groups. In addition, the presence of positively-charged, acidic, and sulfur-containing amino acids improved the ability of antioxidant protein hydrolysates to scavenge free radicals [74]. For instance, Chunkao et al. [21] found that mung bean peptides having “Leu-Leu-Gly-Ile-Leu, Leu-Leu-Leu-Leu-Gly, Pro-Ala-Ile-Asp-Leu, and Pro-Ala-Ile-Asp-Leu and Ala-Ile-Val-Ile-Leu” amino acids sequences which had the highest DPPH (81.27%), FRAP (0.05 mM/mg), HRSA (EC_50_: 0.09 mM) and SRSA (EC_50_: 0.07 mM), respectively. Wali et al. [67] mentioned that different amino acid chains form with varying lengths and compositions. The varied forms play different roles in peptide function, such as a significant role in antioxidant activity. Furthermore, the presence of hydrophobic amino acids inside the peptides causes them to increase their hydrophobic solubility and leads to easier interaction among peptides and donation of protons to radical species [46]. From pinto bean protein hydrolysate, Ngoh and Gan [38] found six sequences for antioxidant peptides, and these peptides’ fraction is <3 kDa. These sequences are “Ala-Cys-Ser-Asn-His-Ser-Pro (1420.64 Da), Pro-Leu-Pro-Leu-His-Met-Leu-Pro (916.52 Da), and Pro-Pro-His-Met-Leu-Pro” (690.35 Da), which displayed the highest FRAP and ABTS, 81 mM, 42.2%, respectively. According to Li et al. [75], increased hydrophobic amino acid content and their location at the third position adjacent to the “C-terminus or the N-terminus” of the sequence were favorable to antioxidant activity. The His-containing peptide could donate protons and scavenge free radicals owing to the imidazole ring. In addition, due to the existence of the phenolic group, F (Phe) might serve as a hydrogen donor, while the SH group in C (Cys) could interact with radicals directly. Furthermore, amino acid residues such as cysteine, histidine, and methionine are essential to the radical scavenging activity of peptides that enhance antioxidant activities due to their unique structural characteristics. “Cysteine” donates sulfur hydrogen; the imidazole group in “histidine” has a proton-donation ability, while “methionine” is prone to oxidation of the methionine sulfoxide [38].

#### 3.1.2. ACE-Inhibitory Peptide

**Figure 2:**
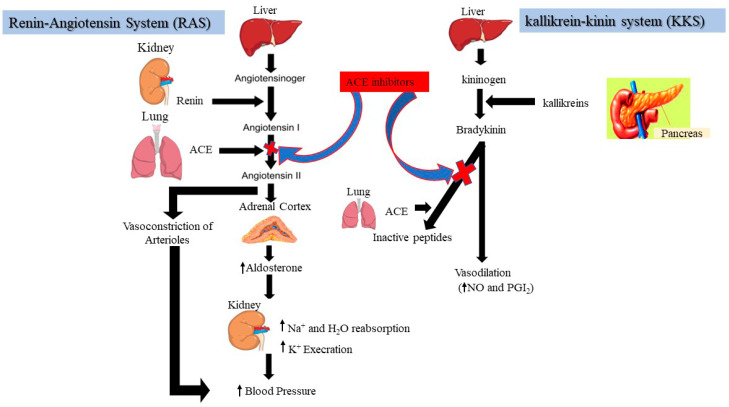
Schematic representation of the role of the renin-angiotensin system (RAS) and the kallikrein-kinin system (KKS) in blood pressure regulation. (Na^+^: Sodium; K^+^: Potassium; NO: Nitric Oxide; PGI2: Prostaglandins 2.

Hypertension, a global issue affecting one-fourth of the world’s adults, is the leading cause of heart disease [76]. The two of the most powerful systems for maintaining blood pressure regulation in humans via the angiotensin-I converting enzyme (ACE) are the renin-angiotensin system (RAS) and the kallikrein-kinin system (KKS), as shown in Figure 2. Angiotensinogen is a substrate for renin to produce angiotensin (I), which is then converted into potent vasoconstrictor angiotensin (II) by ACE, causing raised blood pressure [28,29,30,31,32,33,34,35,36,37,38,39,40,41,42,43,44,45,46,47,48,49,50,51,52,53,54,55,56,57,58,59,60,61,62,63,64,65,66,67,68,69,70,71,72,73,74,75,76,77]. In this context, angiotensin (II) raises oxidative stress when blood pressure is high because it interferes with several of the cell’s functions by increasing the creation of intracellular reactive oxygen species [78]. In contrast, ACE inhibition blocks the first step in the renin-angiotensin system (reduction of angiotensin II), resulting in the treatment of hypertension and enhancing the antioxidative defense system [3]. Inhibitors bind strongly to the ACE active site, contending for occupancy with angiotensin (I); as a result, ACE is unable to convert angiotensin (I) to angiotensin II [79]. Many anti-ACE drugs, such as ramparil, lisinopril, zestril, enalapril, captopril, etc., are commonly used as antihypertensive treatments; with adverse side effects, including taste disturbances, cough, angioedema, and skin rashes [80]. Consequently, natural compounds, especially from plant sources with ACE-inhibitory potential, seem to be of ongoing interest as alternatives to synthetic drugs [81]. Studies have shown that legumes contain beneficial nutrients, bioactive peptides, and high-quality proteins, which have been demonstrated as potent in vitro ACE inhibitors [82].

In bioactive environments, ACE inhibitors can reduce angiotensin (II) production and hence reduce hypertension; for instance, ACE inhibitors are produced from peptides derived from various legumes in vitro, as shown in Table 2. Shi et al. [83] found that a peanut peptide with the amino acid sequence Lys-Leu-Tyr-Met-Arg-Pro and a molecular weight of 808.8 KDa had the highest ACE inhibiting activity at 85.77% (IC_50_: 0.0052 mg/mL). According to Puspitojati et al. [84], ACE-inhibitory peptides usually contain hydrophobic amino acids (Val, Trp, Ile, Phe, Met, Tyr, and Ala) or (Pro) at the C terminal or positively charged amino acids (Lys and Arg). The differences in ACE-inhibitory activity can be attributed to a variety of factors, including the type of legumes, protein extraction technique, type of enzyme used for proteolysis, hydrolysis conditions (temperature, pH, substrate/enzyme ratio, time), and the analytical approach used to determine ACE-inhibitory activity [79]. Figure 1 shows the main steps for preparing and identifying ACE peptides from natural protein sources. Jakubczyk et al. [80] have determined that the peptide fraction III (3.5–7 kDa) of bean protein hydrolysate under conditions of 3 h and a temperature of 22 °C was “Ile-Asn-Glu-Gly-Ser-Leu-Leu-Pro-His” and “Phe-Val-Val-Ala-Glu-Gln-Ala-Gly-Asn-Glu-Glu-Gly-Phe-Glu”, which showed the highest ACE-inhibitory activity (IC_50_: 0.20 μg/mL).

Additionally, Sonklin et al. [15] reported that the ACE inhibition activity of the mung bean hydrolysate was highest in peptide fractions with low MW (<1 kDa, IC_50_: 0.50 mg/mL) when compared to other peptide fractions. The high ACE inhibition activity was due to the lower MW peptides bound to active ACE sites more easily than higher MW peptides after ultrafiltration separation of the protein hydrolysate, resulting in increased ACE inhibition activity. Similarly, Gupta and Bhagyawant [88] found that C. arietinum produced by Alcalase enzyme at 60 min had the highest ACE-inhibitory activity (IC_50_: 0.182 mg/mL) compared to flavourzyme at 100 min (IC_50_: 0.365 mg/mL). The most increased ACE-inhibitory activity is because the peptides made by alcalase are resistant to gastrointestinal proteases, thus absorbed in the small intestine, suggesting that these peptides could be used in the food industry to help people with high blood pressure. In addition, Jakubczyk and Baraniak [37] also demonstrated that peptide fraction (F8B) of pea protein hydrolysate had the highest ACE-inhibitory activity (IC_50_: 0.073 mg/mL) and was identified as these sequences “Gly-Gly-Ser-Gly-Asn-Tyr, Asp-Leu-Lys-Leu-Pro, Gly-Ser-Ser-Asp-Asn-Arg, and His-Asn-Thr-Pro-Ser-Arg”. In this context, the C-terminal amino hydrophobic or aromatic residues significantly impact ACE binding, with proline being the most preferred for high ACE-inhibitory activity; consequently, there is a relationship between ACE-inhibitory peptide activity and structure [23].

Moreover, Hanafi et al. [4] mentioned that the hydrophobicity of the C-terminal amino acids and three-dimensional chemical characteristics significantly impacted ACE-inhibitory activity, demonstrating that the amino acids with the highest volume and hydrophobicity have the strongest ACE-inhibitory activity. Certainly, ACE-inhibitory peptides produced from legume proteins have gained a lot of interest recently, and their ability to prevent hypertension in vitro and in vivo has been thoroughly investigated. Therefore, the use of natural components, such as bioactive legume peptides, to suppress ACE activity has been implemented in studies on cardiovascular problems, particularly hypertension [81].

#### 3.1.3. Anti-Inflammatory Peptide

Inflammation is the immune system’s reaction to adverse stimuli, including infection, tissue damage, or toxic substances. It aims to eliminate pathogenic microorganisms or irritants and promote tissue repair [89]. Nitric oxide (NO) and prostaglandin E2 (PGE2) are pro-inflammatory mediators that are released by microglia in response to inflammatory stimuli through the activation of nuclear factor (NF)-B, which ordinarily activates a protective response in the central nervous system (CNS) to eliminate pathogens and infected cells [90]. There are two lines of defence. The first one is acute inflammation, which happens right after inflammatory response induction and temporarily activates cellular and molecular activities and interactions to stop the spread of infection or damage. The second line is chronic inflammation linked to a higher risk of developing chronic diseases and disorders, including type cancer, 2 diabetes, asthma, and inflammatory bowel disease [91]. 

Some of these diseases are related to inflammation, insulin resistance, and lipotoxicity caused by an excess of adipose tissue (obesity and overweight). Depending on the facts of each case, it is necessary to use drugs with side effects and unfavourable effects. Therefore, pro-inflammatory cytokine inhibitors have been evaluated as potential anti-inflammatory medication options. In contrast, the rise in inflammatory disorders has prompted the search for proteins and peptides with anti-inflammatory effects [92]. In this case, one technique is to research possible compounds, such as plant proteins, that have been studied as a source for bioactive peptides with anti-inflammatory activity agents [93]. Hydrolysates and peptides generated from legumes can have various biological effects, including anti-inflammatory, antioxidant, antihypertensive, anticancer, and immunomodulatory properties [6]. A few particular peptides, such as lunasin, a 43-amino acid chemopreventive peptide extracted from soybean, barley, wheat, rye, and triticale, have been identified as anti-inflammatory peptides from various plant protein hydrolysates [92]. Indrati et al. [94] mentioned that bioactive legume peptides, especially soybeans and *Phaseolus vulgaris* L., can control a variety of inflammatory indicators, including prostaglandin E2 (PGE2), nitric oxide (NO), induced nitric oxide synthase (iNOS), cyclooxygenase 2 (COX2), cytokines, and chemokines.

Additionally, Montserrat-de la Paz et al. [95] reported that lupin protein hydrolysate hydrolyzed by alcalase reduced the levels of nitric oxide and reactive oxygen species in cells and crossed the Caco-2 monolayer of the human intestinal tract to exert anti-inflammatory activity in macrophages located in the basement region by production the pro-inflammatory cytokines and lowering mRNA levels in the vitro study. López-Barrios et al. [96] reported anti-inflammatory activity of *Phaseolus vulgaris* L. peptides was low molecular weight (MW) and contained three to eleven amino acids. Similarly, Garcia-Mora et al. [30] found that pinto beans produced by Alcalase enzyme at 120 min had a higher concentration of small peptides (<3 kDa) with anti-inflammatory activity (28–16%). Moreover, Cruz-Chamorro et al. [97] mentioned that the lupin protein hydrolysate (LPHs) decreased human peripheral blood mononuclear cells proliferation (PBMCs) and the levels of T helper cells (Th1, Th9 and Th17) pro-inflammatory cytokines without being cytotoxic. This improves the anti-inflammatory/pro-inflammatory cytokine balance and reduces T-cell inflammatory responses. High molecular weight peptide fraction (5–10 KDa) from soybean protein hydrolysate showed the best anti-inflammatory activity by reducing the secretion of nitric oxide (NO) and prostaglandin E2 (PGE2) in RAW 264.7 cells [98].

### 3.2. In Vivo Study on Antioxidants, ACE-Inhibitory, and Anti-Inflammatory Peptides

#### 3.2.1. Antioxidant Peptides

The production of antioxidant peptides from several food-derived protein sources through chemical and enzymatic hydrolysis and microbial fermentation has been suggested as an ingredient for producing foods that promote good health [49], as mentioned above. All the antioxidant methods are based on in vitro reactions. While they are helpful for initial screenings of potential antioxidants, more research is needed to confirm the accurate antioxidant capacity of peptides and proteins by measuring antioxidant molecules in plasma or tissues after in vivo assays have been used to verify in vitro results [62]. To date, there have been few studies on the antioxidant impact of legume sources in vivo studies. A peptide sequence (Val-Glu-Leu-Val-Gly-Pro-Lys), recognized as an antioxidative peptide, was discovered by Gomes et al. [99] in bean protein hydrolysate. They found that this peptide lowered MDA levels more than a standard control diet (NC) (3.3 µmol/L) and an atherogenic diet (AD) (3 µmol/L) when combined with an atherogenic diet (APH) (2.7 µmol/L) in male adult BALB/c mice. This was because of the antioxidant peptide and hydrolysate of beans, which prevent lipid peroxidation brought on by an atherogenic diet and prevent free radicals. In addition, He et al. [100] also demonstrated that peptides of rapeseed protein hydrolysate (Leu-Tyr, Arg-Ala-Leu-Pro, and Gly-His-Ser) with a dose of 30 mg/kg peptide increased the superoxide dismutase (SOD) activity (280, 230, 250 U/mL) and decreased the Malondialdehyde(MDA) content (11, 10, 9 nmol/mL) in the plasma of rats compared with the control group,(200 U/mL and 12.5 nmol/mL). These peptides exhibited antioxidant properties that prevented the formation of free radicals and the oxidation of unsaturated fatty acids in vivo. The lengths, sequences, and compositions of amino acid chains come in different forms and played vital roles in the mechanism of antioxidants [67].

#### 3.2.2. ACE-Inhibitory Peptides

##### Toxicity

After gastrointestinal digestion or enzymatic proteolysis, legume protein generates bioactive peptides with various actions, including antioxidative, blood pressure-lowering, and anti-inflammatory effects [101]. Compared to small molecules, peptides have many advantages, including low production costs, high biological activity, and excellent penetration and specificity [102]. Natural peptides play an essential role in treating and preventing several diseases, and they are also widely employed in everyday life, from food production to the cosmetic industry. However, before adopting peptides biologically for therapeutic or food production purposes, the immunogenicity and toxicities of peptides must first be assessed because they have low stability with them [103]. In this context, adding D-amino acids (which make peptides protease-resistant), cyclization, modifying the backbone chemistry, and incorporating α-aminoxy amino acids are all strategies to improve peptide stability [104,105]. In addition, various in silico technologies can predict peptide immunogenicity, yet there is almost no method or approach to predict peptide toxicity. Moreover, computational methods for forecasting peptide toxicity save money and time and make it easier to create better therapeutic peptides with lower toxicity while maintaining functionality [102].

A toxic sample may induce unwanted side effects during tests; thus, an understanding of the toxicity status of the test drug is warranted. A living body is complex, with thousands of cross-organs biochemical interactions co-occurring. In addition, toxicity may cause unpredictably adverse side effects that affect actual efficacy, resulting in underestimated or overestimated biological effectiveness [101]. To date, limited studies have been published on the toxicity of legume proteins and peptides. In the study on winged bean seed (WBS) hydrolysate by Chay et al. [101]; after oral administration of 150 and 300 mg/kg of body weight for 4 h, the highest in vivo blood pressure reductions were −14.8 and −19.3 mmHg, respectively. In addition, WBS peptides were shown for their in vitro ACE-inhibitory properties, such as “Arg-Gly-Val-Phe-Pro-Cys-Leu-Lys, Thr-Gln-Leu-Asp-Leu-Pro-Thr-Gln, Glu-Pro-Ala-Leu-Val-Pro, Met-Arg-Ser-Val-Val-Thr, and Asp-Met-Lys-Pro”. However, according to the following observations, such as weight and biochemical parameters (kidney, liver, and testis), the toxicity status showed that these peptides are toxic and unfit for testing in animal models. In this context, the sample in vitro potency may not accurately reflect its in vivo activity, as the peptides failed to display antihypertensive activity in rats despite substantial inhibitory activities observed in vitro.

In contrast, there have been few studies on the antihypertensive impact of legume sources in SHR without evaluating the toxicity status [33]. Li et al. [106] mentioned that the toxicity of dietary protein-derived peptides had not been reported, even at extremely high dosages used in animal studies and human clinical trials, which is a significant benefit over typical drugs that can cause a variety of side effects. Table 3 shows the impact of ACE peptides on legume protein hydrolysates in spontaneously hypertensive rats (SHRs) in the short and long term. Some trials used a short-term study to see whether the peptides could lower blood pressure in the SHR in vivo before moving on to the more costly and time-consuming long-term study [107]. The main target of the mechanisms of antihypertensive peptides is angiotensin-converting enzyme (ACE), the primary enzyme in the renin-angiotensin II-aldosterone pathway, which also catalyzes the formation of angiotensin II to raise blood pressure. Additional targets of antihypertensive mechanisms of the peptides have also been identified, such as improving NO-mediated vasodilation, enhancing endothelial function, and up-regulating ACE2 expression and gut microbiota [108]. For instance, Aluko et al. [109] discovered that the yellow pea protein hydrolysate had two peptides sequences; “Ile-Phe-Glu-Asn-Leu-Gln-Asn (877 Da)” and “Phe-Glu-Gly-Tyr-Val-Phe-Glu-Asn-Gly (999 Da)”, which had better ACE-inhibitory activity. “Ile-Phe-Glu-Asn-Leu-Gln-Asn” had a higher lowering effect of SBP (−30 mmHg) than did “Phe-Glu-Gly-Tyr-Val-Phe-Glu-Asn-Gly” (−25 mmHg). Low molecular weight peptides penetrate the ACE-active site more profoundly and inhibit ACE activity more effectively than high molecular weight peptides [110]. In addition, Li et al. [107] reported similar results on pea protein hydrolysate (PPH), with ACE inhibition of 19%. As a result, after oral administration to SHR, the capacity of the PPH to interact in vivo and the reduction in SBP was −19 mmHg after 4 h and −13 mmHg after 8 h. This may be because specific inactive peptides in the PPH preparation are converted to active peptides after passing through the gastrointestinal tract (GIT), which are then absorbed through the blood circulation system. Most antihypertensive peptides were small and had a high proportion of aromatic and hydrophobic amino acid residues. As a result, the antihypertensive properties of peptides are structural and dose-dependent [111,112].

##### Antihypertensive

- The Effect of the Peptide on Aorta Histological

The aorta is a dynamic structure that can maintain conditions for optimal mechanical operation by continuous turnover of its internal structure. The aorta is the first arterial segment of systemic blood circulation, directly related to the heart, and is the largest artery in the human body. It comprises the ascending aorta, descending aorta, and abdomen (abdominal aorta) [113]. Aneurysms of the aorta between the iliac bifurcation and the diaphragm are known as abdominal aortic aneurysms (AAAs). They can cause an aortic rupture with fast exsanguination into the retroperitoneum or abdominal cavity if left untreated, which is usually fatal, compared to those of the thoracic aorta, which occur when the usual diameter of the artery increases by 50%. This is due to the aortic wall’s intrinsic weakness [114,115]. Dale et al. [114] mentioned that approximately 15,000 people in the United States die yearly due to aortic aneurysm rupture. The renin-angiotensin system (RAS) plays a vital role in developing thoracic aortic aneurysms (TAAs) and AAAs; thus, pharmaceutical ACE inhibition reduces the risk of aneurysm formation. In addition, several animal studies have shown ACE medications to have beneficial effects against AAAs compared to one non-randomized study that found that an ACE inhibitor inhibited TAA growth in patients with Marfan syndrome [116]. In contrast, Angiotensin II production has been demonstrated in tissues such as the vascular wall and kidney. For instance, captopril, an ACE blocker, is thought to exert an antihypertensive effect by inhibiting ACE in the vascular wall, kidney, brain, and lung [117]. They have shown that the systolic blood pressure (SBP) and ACE activity in the aorta, lung, and kidney, responsible for hypertension, were lower after the feeding of sour milk to SHR rats. It has been shown that peptides isolated from milk (Val-Pro-Pro and Ile-Pro-Pro) were absorbed directly without being degraded by digestive enzymes, reached the abdominal aorta, inhibited the ACE enzyme, and showed antihypertensive effects in SHRs. To date, there are no studies on legume peptides’ effects on the aorta, but a few studies are available from different sources, such as those mentioned above.

- The Effect of the Peptide on Kidney: Functional and Histological

Chronic kidney disease (CKD) is a condition that can lead to kidney failure, cardiovascular disease (CVD), and death [118]. It is a leading cause of morbidity and mortality worldwide, affecting 600 million people, and is characterized by a gradual loss of kidney function [119,120]. In addition, hypertension and diabetes are closely linked to CKD, and natriuretic peptide has been found to reduce blood pressure and expand plasma volume. Patients with CKD complicated by impaired kidney function have higher plasma atrial (ANP) and brain natriuretic (BNP) peptides. However, the association between the plasma level of ANP or BNP and the future development of CKD is unknown [121].

Furthermore, when the kidneys are injured, the excretion of urea and creatinine, which are waste products of protein metabolism, is impaired. Creatinine is a compound found in skeletal muscle tissue and is highly useful in measuring renal function. The increased creatinine and urea levels in the blood are related to the retention of waste products in the body, suggesting kidney impairment defined by a lower glomerular filtration rate (GFR) [122]. In addition, electrolytes, total protein, and albumin are indicators of kidney function [123]. Many factors, including hyperactivity of ACE and renin in the renin-angiotensin system (RAS), atrial and brain natriuretic peptides, cyclooxygenase-1 (COX-1), and calmodulin phosphodiesterase 1, are linked to kidney damage. Additionally, these factors are related to hypertension [111,124].

Therefore, legumes are widely acknowledged for contributing to a well-balanced diet and helping to avoid non-communicable disease progression, such as CVD. Based on this, dietary protein restriction is a successful strategy for reducing CKD signs and symptoms, although it is undesirable to many patients and can lead to protein malnutrition [125]. Therefore, the bioactive peptides produced by enzymatic hydrolysis of food proteins have been proven to have promising effects on human health and illness, and the synthesis of prospective peptides should be based on the characteristics of critical organ functions, such as the kidneys, in this case [118]. However, studies examining the impact of plant proteins on renal disease are mostly limited to soy protein from legumes, proposed as a partial replacement or a safe alternative for animal protein-based diets in CKD [126]. Meanwhile, Li et al. [106] discovered that protein hydrolysate from yellow peas could be a natural remedy for CKD and high blood pressure.

Additionally, according to levels of urea, creatinine, and COX-1, Hidayat et al. [118] found that green pea protein hydrolysate hydrolyzed by bromelain was the most efficient in enhancing kidney function in Wistar rats exposed to CP-induced nephrotoxicity. There are also only a few studies of the peptide on kidney impact from legume sources. Chay et al. [101] have shown that the peptide identified from winged bean seed hydrolysate had a more substantial toxicity effect in males than female rats through an impact on kidney function. They reported that electrolytes such as chloride, potassium, sodium, and albumin levels were outside of normal physiological ranges at the dosage of 10 mg/kg in male rats, indicating impaired kidney function. Compared to the females, the electrolytes were normal, but creatinine levels were lower at the dosages of 10 and 100 mg/kg. Wallig et al. [127] found that because of its function of filtering and excreting drug metabolites from the body, the kidney is frequently exposed to high metabolites and becomes a target for toxicity. In addition, low protein and albumin levels indicate kidney malfunction because they represent the kidney’s failure to retain produced protein, resulting in protein loss in the urine [123].

#### 3.2.3. Anti-Inflammatory Peptides

Bioactive peptides are an important class of functional food ingredients that may affect human health and their nutritional value, including antihypertensive, anti-inflammatory, antioxidant, chemopreventive, and anti-diabetic properties [128]. Several bioactive peptides from various sources have been investigated as anti-inflammatory in animal models (in vivo studies). This allows the evaluation of the effects of these compounds in more complex physiological conditions than in vitro studies [89]. For example, Gomes et al. [99] mentioned that bean protein hydrolysate with an atherogenic diet showed lower HDL cholesterol (47 mg dL^−1^) than a standard control diet (NC) (68 mg dL^−1^) and atherogenic diet (AD) (55 mg dL^−1^) in male adult BALB/c mice. This resulted in preventing inflammation and endothelium dysfunction and a decline in oxidative stress, showing an adjuvant effect on lowering atherogenic risk. In addition, Kovacs-Nolan et al. [129] discovered that the soybean protein hydrolysate had a “Val-Pro-Tyr” peptide sequence which showed an anti-inflammatory effect by reduced MPO activity, decreased gene expression of the pro-inflammatory cytokines TNF-α, IL-6, IL-1β, IFN-γ, and IL-17 in the colon, improved colon histology, and reduced DSS-induced colitis symptoms and weight loss in vivo in a mouse model of Dextran sodium sulfate DSS-induced colitis. To date, few in vivo studies on the anti-inflammatory impact of legume sources have been performed.

## 4. Future Trends

Although this review has highlighted the antioxidant, ACE-inhibitory, and anti-inflammatory peptides’ impact, which was isolated from legume protein hydrolysate in the in vitro and in vivo studies, there is still more to be explained about this issue. Therefore, additional research, including animal models such as spontaneously hypertensive rats, is needed to investigate the potent peptides’ in vivo behavior. In addition, further research is required to investigate the antioxidant activities in vivo. Furthermore, the toxicity of legume protein and peptides as natural agents for hypertension treatment should be studied further. More research is needed to determine the inflammatory effect of the legume’s peptide on the aorta and kidney histology. In addition, the determination of the peptides responsible for the high ACE-inhibitory activity, as well as enzymes that could potentially release more potent peptides, is desired. Future research should focus on in vivo and molecular docking studies to fully understand the peptide’s mechanism of action and the development of hypertension and inflammatory drugs. Increased research into legume-derived peptides in antihypertensive therapy may significantly impact the pharmaceutical and nutraceutical industries by improving patient quality of life or lowering hypertension risks.

## 5. Conclusions

This review discusses legume protein hydrolysates, in vitro and in vivo studies of antioxidant ACE-inhibitory and anti-inflammatory peptides, and the structure-function of legume protein hydrolysates. This review found that the research on a legume protein hydrolysate’s antioxidant ACE-inhibitory and anti-inflammatory peptide is still limited to in vitro trials, with only a few in vivo. Low molecular weight, short chain sequence, and hydrophobic amino acids like Pro, Val, Leu, Ala, Tyr, Trp, Phe, Met, and Gly are the basis for the antioxidant, antihypertensive, and anti-inflammatory bioactivity of legume peptides. In vitro studies showed that protein hydrolysate or peptides from legumes have higher significant ACE-inhibitory and antioxidant activities effects. Studies have shown that the legume peptides, which possess antihypertensive activity, can reduce SBP in the SHR in in vivo experiments. These peptides’ anti-ACE drug side effects will be considerably less than those of commercial anti-ACE pharmaceuticals because they are sourced from natural sources (legume sources). In this sense, legume peptides could be used as natural antioxidants, antihypertensive, and anti-inflammatory peptides in food and functional items.

## Figures and Tables

**Table 1:** In vitro antioxidant activities of legume protein hydrolysates and peptides.

Legumes	Enzyme	Antioxidant Activities of Hydrolysate	Amino acid Sequences with MW	Antioxidant Activities of Sequences	IC_50_ of Sequences	Reference
Chickpea	Alcalase	DPPH	“Asn-Arg-Tyr-His-Glu (717.37Da)”	45.33%	* NR	[69]
		HRSA	* NR	60.09%	* NR	
		SRSA	* NR	79.81%	* NR	
		Fe^2+^ chelating activity	* NR	60.09%	* NR	
	Alcalase + Flavorzyme	DPPH	“Arg-Gln-Ser-His-Phe-Ala-Asn-Ala-Gln- Pro (1155 Da)”	41.3%	* NR	[68]
		HRSA	* NR	74.6%	* NR	
		ABTS	* NR	0.967 mmoL/L	* NR	
		Reducing power	* NR	0.247	* NR	
	Alcalase	DPPH 79%	“Asp-His-Gly (327 Da)”	51.66%	* NR	[70]
			“Val-Gly-Asp-Ile (402 Da)”	67.32%	* NR	
		Reducing power 1.6	* NR	* NR	* NR	
		Fe^2+^ chelating activity 50.45%	* NR	* NR	* NR	
	Neutrase	DPPH	“Leu-Thr-Glu-IIe-IIe-Pro”.	* NR	0.24 mg/mL	[67]
		HRSA	* NR	* NR	0.57 mg/mL	
Lentil	Pepsin + trypsin	DPPH	“Ala-Leu-Gly-Pro-Val-Met (587.31 Da)	63%	* NR	[65]
		HRSA	* NR	40%	* NR	
	Alcalse	DPPH 25.3%	* NR	* NR	* NR	[19]
Soy	Proteinase	DPPH 5.30 mg/ml	* NR	* NR	* NR	[17]
		FRAP 1.17	* NR	* NR	* NR	
Peanut	Alcalase	Reducing power	“Thr-Pro-Ala (286 kDa)”	2.0	* NR	[53]
			“Ile/Leu-Pro-Ser (315 kDa)”	2.0	* NR	
			“Ser-Pro (202 kDa)”	1.8	* NR	
	Papain	DPPH 74.88%	* NR	* NR	* NR	[71]
		HRSA 82.06%	* NR	* NR	* NR	
		Reducing power 0.671	* NR	* NR	* NR	
Pea	Alcalase	DPPH F1-2(37.94%)	<3 kDa: F1-2			[72]
		HRSA F1-2(28.43%)	“Tyr-Ser-Ser-Pro-Ile-His-Ile-Trp”	* NR	* NR	
			“Ala-Asp-Leu-Tyr-Asn-Pro-Arg”	* NR	* NR	
			“His-Tyr-Asp-Ser-Glu-Ala-Ile-Leu-Phe”	* NR	* NR	
Lupin	Alcalase	ABTS 2.7 μmoL Trolox eq./mg	* NR	* NR	* NR	[66]
		ORAC 3.8 μmoL Trolox eq./mg	NR	* NR	* NR	
Black bean	Pepsin	DPPH 45.15%	* NR	* NR	* NR	[73]
	Alcalase	ABTS 63.56%	* NR	* NR	* NR	
Pinto beans	Protamex	ABTS 53.3%	<3 kDa:	42.2%	* NR	[38]
		FRAP 3.71 mM	“Pro-Pro-His-Met-Leu-Pro (690.35 Da)”	0.81 mM	* NR	
			“Pro-Pro-Met-His-Leu-Pro (690.35 Da)”	* NR	* NR	
			“Pro-Leu-Pro-Pro-His-Met-Leu-Pro (900.49 Da)”	* NR	* NR	
			“Pro-Leu-Pro-Leu-His-Met-Leu-Pro (916.52 Da)”	* NR	* NR	
			“Ala-Cys-Ser-Asn-His-Ser-Pro-Leu-Gly-Trp-Arg-Gly-His (1420.64 Da)”	* NR	* NR	
			“Leu-Ser-Ser-Leu-Glu-Met-Gly-Ser-Leu-Gly-Ala-“Leu-Phe-Val-Cys-Met (1656.79 Da)”	* NR	* NR	
Mung bean	Pancreatin	DPPH	“Leu-Leu-Gly-Ile-Leu”	81.27%		[21]
			“Pro-Ala-Ile-Asp-Leu”	46.63%		
		HRSA	“Leu-Leu-Gly-Ile-Leu”		0.37 mM	
			“Pro-Ala-Ile-Asp-Leu”.		0.09 mM	
		SRSA	“Pro-Ala-Ile-Asp-Leu. and Ala-Ile-Val- Ile–Leu”	* NR	0.07 mM	
		FRAP	“Leu-Leu-Leu-Leu-Gly”	0.05 mM/mg	* NR	
Dark red kidney	Pepsin	DPPH 81.41%	* NR	* NR	* NR	[42]

* NR: Not Reported; DPPH: 2,2-diphenyl-1-picrylhydrazyl; FRAP: Ferric reducing antioxidant power; SRSA: Superoxide radical scavenging activity; HRSA: Hydroxyl radical scavenging activity; ABTS: 2,2-azino-bis-3-ethylbenzothiazoline-6-sulfonic Acid; ORAC: Oxygen radical absorbance capacity.

**Table 2:** In vitro inhibition of ACE of legume protein hydrolysates and peptides.

Legumes	Enzyme	ACE-Inhibitory Activity of Hydrolysate (%)/IC_50_	Potential Sequences with MW	ACE-Inhibitory Activity of Sequences (%)/IC_50_	Reference
Soybean	Alcalase	* NR	<3 kDa.		[23]
			“Ile-Tyr”	93.30%/0.53 μM	
			“Ile-Val-Val-Phe”	74.25%/0.27 mM	
			“Leu-Val-Phe”	66.18%/0.36 mM	
			“Trp-Met-Phe”	52.77%/0.55 mM	
			“Leu-Phe-Leu-Leu”	41.87%/0.72 mM	
			“Phe-Phe”	41.85%/0.73 mM	
	Alcalase	0.014 mg/mL	“Pro-Ser-Leu-Aeg-Ser-Tyr-Leu-Ala-Glu (1035.16 Da)”	99.31%/532 μM	[4]
			“Glu-Ala-Gln-Arg-Leu-Leu-Phe (876.02 Da)”	94.19%/878 μM	
			“Arg-Gly-Gln-Val-Leu-Ser (658.75 Da)”.	90.40%/993 μM	
			“Phe-Ile-Thr-Ala-Phe-Arg (753.90 Da)”	101.51%/1342 μM	
			“Pro-Asp-Arg-Ser-Ile-His-Gly-Arg-Gln-Leu-Ala-Glu (1378.51 Da)”.	92.92%/1552 μM	
Soy	Chymotrypsin + Thermolysin	35 mg/mL	“Leu-Trp”	1.1 µ/mol L	[85]
			“Val-Trp”	3.5 µ/mol L	
			“Leu-Tyr”	5.2 µ/mol L	
			“Val-Ty”	9.4 µ/mol L	
	Proteinase	20.31%	* NR	* NR	[17]
Pea	Chymotrypsin + Thermolysin	33.5 mg/mL	“Leu-Trp”	1.1 µ/mol L	[83]
			“Val-Trp”	3.5 µ/mol L	
			“Leu-Tyr”	5.2 µ/mol L	
			“Val-Tyr”	9.4 µ/mol L	
	Pepsin + Pancreatin + Amylase	0.72 mg/mL	Fraction (F8)-(F8B)	0.0014 mg/mL 0.073 mg/mL	[37]
			“Gly-Gly-Ser-Gly-Asn-Tyr”	* NR	
			“Asp-Leu-Lys-Leu-Pro”	* NR	
			“Gly-Ser-Ser-Asp-Asn-Arg”	* NR	
			“Met-Arg-Asp-Leu-Lys”	* NR	
			“His-Asn-Thr-Pro-Ser-Arg’	* NR	
Pigeon pea	Pepsin + Pancreatin	77.82%	* NR	* NR	[3]
	Pancreatin	74%	* NR		
Bean	Pepsin + Pancreatin + Amylase	* NR	3.5–7 kDa “Ile-Asn-Glu-Gly-Ser-Leu-Leu-Pro-His”	0.20 μg/mL * NR	[80]
			“Phe-Val-Val-Ala-Glu-Gln-Ala-Gly-Asn-Glu-Glu-Gln-Phe-Glu”	* NR	
Lima bean	Pepsin–Pancreatin	* NR	>3 KDa	60.15%/172.62 μg/mL	[82]
Chickpea	Alcalase	52.22 µg/mL	* NR	* NR	[22]
Lupin	Pepsin	226 µg/mL	* NR	* NR	[79]
Mung bean	Alcalase	50.20%/20.37 μg/mL	<3 kDa	71.22%/4.66 μg/mL	[86]
	Bromelain	0.69 mg/mL	Fraction (F4) < 1 kDa	0.50 mg/mL	[15]
			“Tyr-Ala-Asp-Leu-Val-Glu”	ND *	
			“Lue-Arg-Leu-Glu-Ser-Phe”	5.39 µM	
			“Pro-Gly-Ser-Gly-Cys-Ala-Gly-Thr-Asp-Leu”	57.86 µM	
			“Leu-Pro-Arg-Leu”	1912 µM	
Kidney Bean	Alcalase	80%	<1 kDa + 3–5 kDa 1–3 + 3–5 kDa	77% 79.5%	[87]

* ND: Not Detected; * NR: Not Reported.

**Table 3:** In vivo: Effect of ACE peptide of legume protein hydrolysates in spontaneously hypertensive rats (HSRs) as short- and long-term.

Legumes	Enzyme	MW of Peptides	ACE-Inhibitory Peptide (%)/IC_50_	Dose of Sample mg/Kg	Short Term (24 h) (Sample/SBP/ Time/Day) in SHR	Long Term (Sample/SBP/ Time or Day) in SHR	Potential Sequences	ACE-Inhibitory Sequences (%)/IC_50_	Reference
Pea	Thermoase	5 kDa	0.10 mg/mL	100	HTPPI/−25 mmHg/6 h.	1% HTPPI/−17 mmHg/3rd wk.	* NR	* NR	[109]
				100	PPH-5/−36 mmHg/2 or 4 h.	1% PPH-5/−26 mmHg/3rd wk.	* NR	* NR	
	Thermolysin	<3 kDa	19%	100	PPH/−19 mmHg/4 h.	PPH/−29 mmHg/6 week.	* NR	* NR	[107]
	Thermolysin	3 kDa	NR	100	PPH-3/−9 mmHg/2.	* NR	* NR	* NR	[108]
		877 Da	NR	30	IFENLQN/−37 mmHg.	* NR	Ile-Phe-Glu-Asn-Leu-Gln-Asn.	87.54%	
		999 Da	NR	30	FEGTVFENG/−25 mmHg.	* NR	“Phe-Glu-Gly-Tyr-Val-Phe-Glu-Asn-Gly”	76.77%	
Azufrado Beans	Alcalase	3 kDa	3.68 µg/mL or 53.43%	500	BP3/−41 mmHg/3 h.	BP3/−24 mmHg/45 days.	“Lys-Phe-Pro-Try-Val-Lys”		[33]
							“Gly-Ala-Asp-Phe-Aeg-Lys-Lys”		
							“Pro-Gln-Ser-Pro-Cys-Lys-Arg-Val-Asn-Arg-His-Ser”		
Mung bean	Bromelain	<1 kDa	0.69 mg/mL	20	MPH/−19 mmHg/24 h.	* NR	* NR		[15]
			42.90 mg/mL	20	F4-P10 YADLVE/−27 mmHg/24 h.	* NR	“Tyr-Ala-Asp-Lue-Val-Glu.”	* NR	
lima bean	Pepsin–Pancreatin	>3 kDa	60.15% or 172.62 µg/mL	15	* NR	>3 kDa/51%/3 week	* NR	* NR	[82]
Peanut	Alcalase + N120P	<1 kDa (PP-II)	85.77 or 0.091 mg/mL	100 500 1000	P8/145 mmHg/3 h P8/136 mmHg/3 h P8/134 mmHg/3 h	Over 160 mmHg/1 week	Lys-Leu-Tyr-Met-Arg-Pro. (P8).	0.0052 mg/mL	[83]

MW: Molecular weight; SBP: Systolic Blood Pressure; SHR: Spontaneously Hypertensive rat, * NR: Not Reported.
